# Dietary protein intake and risk of ovarian cancer: evidence from a meta-analysis of observational studies

**DOI:** 10.1042/BSR20181857

**Published:** 2018-12-11

**Authors:** Yanyang Pang, Wu Wang

**Affiliations:** 1 Department of Traditional Chinese Medicine, Hainan Medical University, Haikou, Hainan 571101, China; 2 Laboratory of Tropical Biomedicine and Biotechnology and School of Tropical Medicine and Laboratory Medicine, Hainan Medical University, Haikou, Hainan 571101, China

**Keywords:** Meta-analysis, Ovarian cancer, Protein intake

## Abstract

The association between dietary protein intake and ovarian cancer had been inconsistent in the previous epidemiological studies. The aim of the present study was to identify and synthesize all citations evaluating the relationship on ovarian cancer with protein intake. The search included PubMed, Embase, and Web of Science from inception to June 2018. Two authors independently selected studies, extracted data, and assessed risk of bias. Relative risk (RR) and 95% confidence interval (95%CI) were calculated for relationship between the dietary protein intake and ovarian cancer risk using a random-effects model. Publication bias was evaluated using Egger’s test and Begg’s funnel plots. At the end, ten citations with 2354 patients were included in meta-analysis. Summarized RR with 95%CI on ovarian cancer was 0.915 (95%CI = 0.821–1.021), with no between-study heterogeneity (*I^2^* = 0.0%, *P*=0.708). The results were consistent both in animal protein intake and in vegetable intake on ovarian cancer. Subgroup analysis by study design did not find positive association either in cohort studies or in case–control studies. Egger’s test (*P*=0.230) and Funnel plot suggested no publication bias. Based on the obtained results, we conclude that high dietary protein intake had no significant association on ovarian cancer risk. Besides that, it is necessary to develop high quality, large-scale studies with detailed amount of dietary protein intake for verifying our results.

## Introduction

Ovarian cancer is the most lethal gynecologic cancer. The American Cancer Society estimated 22240 new ovarian cancer cases and 14070 ovarian cancer deaths in 2018 [1]. The prognosis of ovarian cancer remains poor; the 5-year survival rate is approximately 45.6% overall and approximately 25% for stages III and IV disease [2]. Most ovarian cancers are epithelial carcinomas, and its pathogenesis is multi-faceted. Various chemical, physical, biological, and other carcinogenic factors as well as immune function, endocrine, genetic, and spiritual factors are the causes of ovarian cancer [2]. In addition, malnutrition and personal habits are also important causes of ovarian cancer [2]. Therefore, it is important to prevent ovarian cancer.

Previous meta-analyses had suggested that cruciferous vegetables intake [3], flavonoids intake, flavonoid subclasses intake [4], and calcium intake [5] could reduce the ovarian cancer risk. Some studies also indicated that dietary fat intake [6] and egg consumption [7] could increase the risk of ovarian cancer. Therefore, diet is an important aspect to prevent ovarian cancer. Dietary protein intake had produced inconsistent results on ovarian cancer risk [8–10]. The aim of this report was to identify and synthesize all citations evaluating the relationship between dietary protein intake and ovarian cancer risk.

## Materials and methods

### Publication search strategy

A comprehensive literature search was conducted on platforms of PubMed, Embase, and Web of Science. The last search was performed on June 2018. Free words adopted were as follows: (‘protein’ OR ‘nutrient’ OR ‘nutrition’ OR ‘dietary’) AND (‘ovarian cancer’ OR ‘ovarian tumor’). The reference lists of the full-text articles were manually examined to identify any additional publications relevant to our analysis. The language of publications was restrained to English.

### Study selection and data extraction

The inclusion criteria were as follows: (i) observational studies; (ii) evaluating the association between dietary protein intake and ovarian cancer; (iii) odds radio (OR) in case–control studies and relative risk (RR) in cohort studies and their 95% confidence interval (95%CI) for protein were reported in text or could be computed from given data; (iv) reporting the studies on humans; and (v) studies published in English language. The exclusion criteria were as follows: (i) animals study; (ii) letters or case reports; (iii) articles that provided inadequate information of interest or primary data; and (iv) published not in English.

The course of study selection and data extraction was completed by two investigators independently. Excel database was used to extract the following information from included studies: first author’s name, publication year, study design, age, amount of cases and participants, country, protein type, categories of dietary protein, OR/RR with 95%CI on ovarian cancer risk, and adjustment for factors. Any resulting discrepancies were resolved by discussion with the two investigators together.

### Statistical methods

Statistical analyses were performed using Stata version 12.0 (StataCorp LP, College Station, TX). RR and 95%CI were calculated to assess the association between dietary protein intake and ovarian cancer risk [11]. Random-effects model was used throughout the study [12]. Subgroup analyses by protein type, study design, and geographic locations were performed. *P-*values less than or equal to 0.05 were considered as statistically significant. Heterogeneity was examined by Q test and *I^2^* test. *P*<0.1 in Q test or *I^2^* > 50% indicated statistically significant heterogeneity [13]. Publication bias was evaluated using Egger’s test [14] and Begg’s funnel plots [15].

## Results

### Search results and study characteristics

**Figure 1:**
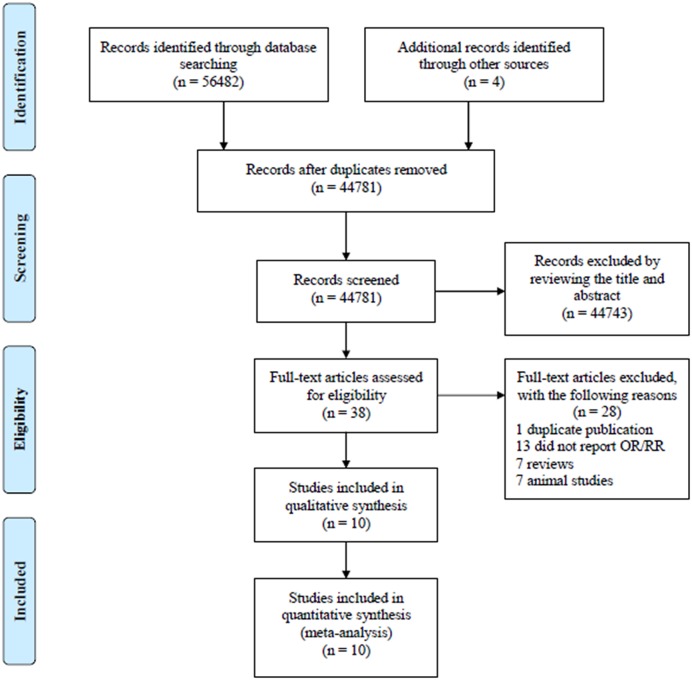
Study selection process for this meta-analysis

A total of 56482 citations were found from the databases and 4 additional records identified through other sources. There were 44781 records reviewed when the duplicates from different databases were removed. Consequently, 44743 citations were removed after viewing title and abstract. Furthermore, 28 citations were removed due to some reasons (Figure 1). At the end, 10 citations [8–10,16–22] with 2354 patients were included in meta-analysis and were from North America or Europe. The basic features of all citations are shown in Table 1.

**Table 1:** Characteristics of the studies about dietary protein intake and ovarian cancer risk

Study (year)	Design	Age	Participants, cases	Country	Protein type	Categories	RR (95%CI)	Adjustment
Byers et al. (1983)[8]	HCC	30–79	1034, 274	United States	Total protein	Tertiles 1	1	Adjusted for age
						Tertiles 2	1.12 (0.68–1.56)	
						Tertiles 3	1.16 (0.74–1.58)	
Kiani et al. (2006)[9]	Cohort	≥25	34192, 71	United States	Total protein	56.2 g/week (continue)	0.83 (0.67–1.04)	Adjusted for age, parity and BMI, and also for age at menopause and hormone replacement therapy in postmenopausal analyses
Kushi et al. (1999)[16]	Cohort	55–69	29083, 139	United States	Total protein, Animal protein, Vegetable protein	Total protein <72.6 g/day 72.6–81.1 81.2–89.9 >89.9 Animal <49.6 g/day 49.6–58.9 59.0–69.0 >69.0 Vegetable <19.0 g/day 19.0–21.6 21.7–24.5 >24.5	Total protein 1 1.04 (0.61–1.77) 1.19 (0.71–1.98) 1.16 (0.69–1.92) Animal 1 1.50 (0.89–2.53) 1.31 (0.77–2.24) 1.32 (0.77–2.24) Vegetable 1 1.48 (0.89–2.47) 1.47 (0.88–2.44) 0.83 (0.47–1.48	Adjusted for age, total energy intake, number of live births, age at menopause, family history of ovarian cancer in a first-degree relative, hysterectomy/unilateral oophorectomy status, waist-to-hip ratio, level of physical activity, cigarette smoking (number of pack-years), and educational level
McCann et al. (2001)[17]	HCC	20–87	1921, 496	United States	Total protein	<56 (g/day) 57–76 77–102 >102	1 1.08 (0.77–1.51) 1.10 (0.74–1.61) 1.20 (0.69–2.08)	Adjusted for age, education, region of residence, regularity of menstruation, family history of ovarian cancer, parity, age at menarche, oral contraceptive use, and total energy intake
McCann et al. (2003)[18]	PCC	40–85	820, 124	United States	Total protein	<65 (g/day) 65–82 82–96 96–117 >117	1 0.73 (0.37–1.44) 0.98 (0.50–1.92) 1.43 (0.73–2.82) 0.80 (0.32-2.00)	Adjusted for age, education, total months menstruating, difficulty becoming pregnant, oral contraceptive use (ever/never), menopausal status and total energy
Pan et al. (2004)[19]	PCC	20–76	2577, 442	Canada	Total protein	Quartile 1 Quartile 2 Quartile 3 Quartile 4	1 0.87 (0.63–1.19) 0.86 (0.62–1.17) 1.00 (0.73–1.35)	Adjusted for 10-year age group, province of residence, education, alcohol consumption, cigarette pack-years, BMI, total caloric intake, recreational physical activity, number of live births, menstruation years, and menopause status
Risch et al. (1994)[10]	PCC	35–79	1014, 450	Canada	Total protein, Animal protein, Vegetable protein	Total protein 40 g/day (continue) Animal 10 g/day Vegetable 10 g/day	Total protein 0.75 (0.56–1.00) Animal 0.96 (0.90–1.02) Vegetable 0.91 (0.789–1.06)	Adjusted for age at diagnosis/interview and the continuous variables age, total daily calorie intake, number of full-term pregnancies, and total duration of oral contraceptive use. Each line in this table represents two individual models
Salazar-Martinez et al. (2002)[20]	HCC	20–79	713, 84	Mexico	Total protein, Animal protein, Vegetable protein	Total protein ≤48 (g/day) 49–69 ≥70 Animal ≤28 (g/day) 29–43 ≥44 Vegetable ≤18 (g/day) 19-27 ≥28	Total protein 1 0.7 (0.37–1.3) 0.88 (0.5–1.53) Animal 1 0.94 (0.52–1.70) 0.68 (0.38–1.22) Vegetable 1 0.80 (0.43–1.47) 0.92 (0.52–1.62)	Adjusted for age, total energy intake, number of live births, recent changes in weight, physical activity, and diabetes
Slattery et al. (1989)[21]	PCC	20–79	577, 85	United States	Total protein	<70.1 (g/day) 70.1–97.3 >97.3	1 1.2 (0.7–2.1) 1.0 (0.5–1.8)	Adjusted for age, body mass index of weight/height^2^, and number of pregnancies. All dietary variables are in separate logistic models
Tzonou et al. (1993)^[^22]	HCC	18–75	389, 189	Greece	Total protein	Highest compared with lowest	0.92 (0.73–1.15)	Adjusted for age, years of schooling, parity, age at first birth, menopausal status as well as for energy intake

Abbreviations: HCC, hospital-based case–control study; PCC, population-based case–control study.

### Meta-analysis

**Figure 2:**
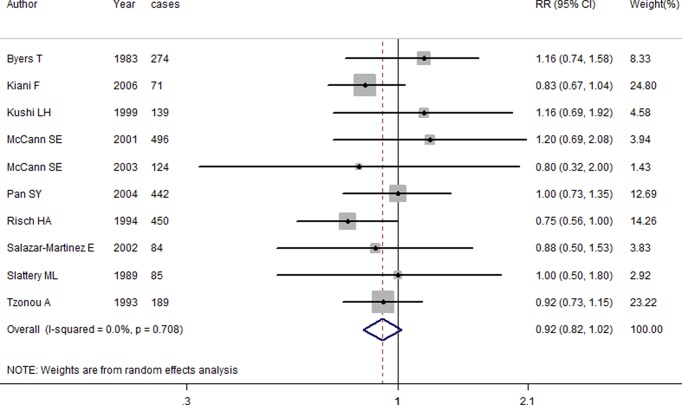
Forest plot of the association between dietary protein intake and ovarian cancer risk

As shown in Figure 2, highest category of dietary protein intake compared with lowest category was not associated with ovarian cancer risk (RR = 0.915, 95%CI = 0.821–1.021), with no between-study heterogeneity (*I*^*2*^ = 0.0%, *P*=0.708). Meanwhile, the relationship was not significant either in vegetable protein intake (RR = 0.906, 95%CI = 0.789–1.040) or in animal protein intake (RR = 0.963, 95%CI = 0.778–1.191). Nine of the ten studies were from North America, the summarized RR (95%CI) was 0.914 (95%CI = 0.807–1.036). The association of subgroup analysis by study design was not significant. The detailed results are shown in Table 2.

**Table 2:** Summary RR and 95%CI of the association about dietary protein intake and ovarian cancer risk

Subgroups	Number of studies	Number of cases	RR	95%CI	Z test	*P* for trend	Heterogeneity test	
							*I^2^*^ (^%)	*P*
Overall	10	2354	0.915	0.821–1.021	1.58	0.114	0.0	0.708
Protein type								
Animal protein	3	673	0.963	0.778–1.191	0.35	0.726	26.2	0.258
Vegetable protein	3	673	0.906	0.789–1.040	1.40	0.160	0.0	0.953
Study design								
Cohort	2	210	0.903	0.679–1.201	0.70	0.483	27.9	0.239
Case–control	8	2144	0.933	0.819–1.063	1.04	0.297	0.0	0.703
PCC	4	1101	0.868	0.714–1.056	1.41	0.157	0.0	0.571
HCC	4	1043	0.988	0.830–1.177	0.13	0.893	0.0	0.635
Geographic locations								
North America	9	2165	0.914	0.807–1.036	1.41	0.159	0.0	0.612
Europe	1	-	-	-	-	-	-	-

Abbreviations: HCC, hospital-based case–control study; PCC, population-based case–control study.

### Publication bias analysis and sensitivity analysis

**Figure 3:**
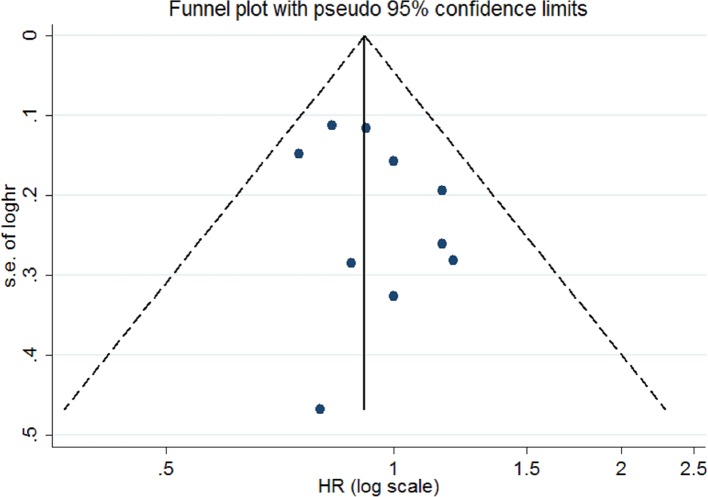
Funnel plots of the association between dietary protein intake and ovarian cancer risk

**Figure 4:**
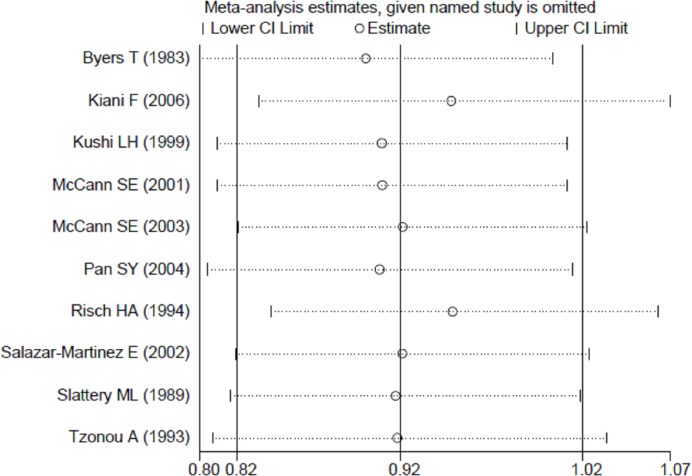
Sensitivity analysis of the association between dietary protein intake and ovarian cancer risk

Publication bias was not found by Begg’s funnel plots (Figure 3), as well as Egger’s test (*P*=0.230). Figure 4 showed that no single study had essential effect on the overall results.

## Discussion

We conducted this first comprehensive meta-analysis to assess the association between dietary protein intake and ovarian cancer risk. Findings from the above results suggested that higher dietary protein intake compared with lower intake had no significant association on ovarian cancer risk. The association was not significant either in cohort studies or in case–control studies. Nine of the ten studies were conducted in North America, resulted a non-significant association between dietary protein intake and ovarian cancer risk.

To our knowledge, protein contains 22 known amino acids. Nine of the 22 are essential amino acids, which cannot be synthesized in the body [23]. Thus, people may obtain these essential amino acids from some levels of foods, such as animal meats, plants such as soy, and dairy products [23]. Protein deficiency can lead to growth retardation, nutritional edema, or may even endanger life [24]. Two previous meta-analyses were conducted to assess whether highest category of dietary protein intake compared with lowest category could reduce the risk of prostate cancer [25] or colorectal cancer [26]. They concluded that higher intake of protein had no relationship either on prostate cancer risk or on colorectal cancer risk. In our report, we did not find reverse association about ovarian cancer with higher animal protein intake or vegetable protein intake, probability due to the small number of studies included for animal protein intake and vegetable protein intake. All these results obtained from our report were consistent with the previous two meta-analyses.

In the overall analysis, there was no between-study heterogeneity as shown in Figure 2. Subgroup analyses by protein type, study design, geographic locations also showed low between-study heterogeneity. Furthermore, no publication bias was found and no single study had essential effect on the pooled results suggested that our results are stable.

Some potential limitations in the present study require attention. First, only English language publications were searched and only English articles were included, therefore, some other language studies were omitted in our analysis. However, no publication bias was found. Second, eight of the ten studies were case–control studies and only two were cohort studies. The retrospective nature of case–control studies and the possibility of bias, recall bias, and confounding factors cannot be excluded. Even so, case–control study was a very important epidemiological approach in the observational study. Otherwise, it is a requirement for evidence from prospective cohort studies. Third, almost all studies included in our analysis were from North America, and the result was consistent with overall pooled result. Therefore, our conclusions may be limited to the North American population and its implications in other populations need to be further investigated. Furthermore, more studies in other populations are wanted to assess dietary protein intake and ovarian cancer risk in the future.

Based on the obtained results, we concluded that high dietary protein intake had no significant association with ovarian cancer risk. Besides that, it is necessary to develop high quality; large-scale studies with detailed amount of dietary protein intake for verifying our results.

## Abbreviations

CI: confidence interval
OR: odds ratio
RR: relative risk
